# Lipopolysaccharide-induced NF-κB nuclear translocation is primarily dependent on MyD88, but TNFα expression requires TRIF and MyD88

**DOI:** 10.1038/s41598-017-01600-y

**Published:** 2017-05-03

**Authors:** Jiro Sakai, Eugenia Cammarota, John A. Wright, Pietro Cicuta, Rachel A. Gottschalk, Ning Li, Iain D. C. Fraser, Clare E. Bryant

**Affiliations:** 1 Department of Veterinary Medicine, University of Cambridge, Cambridge CB3 0ES, United Kingdom; 2 Sector of Biological and Soft Systems, Cavendish Laboratory, University of Cambridge, Cambridge CB3 0HE, United Kingdom; 3 Laboratory of Systems Biology, National Institute of Allergy and Infectious Diseases, National Institute of Heath, Bethesda, MD 20892 USA

## Abstract

TLR4 signalling through the MyD88 and TRIF-dependent pathways initiates translocation of the transcription factor NF-κB into the nucleus. In cell population studies using mathematical modeling and functional analyses, Cheng *et al*. suggested that LPS-driven activation of MyD88, in the absence of TRIF, impairs NF-κB translocation. We tested the model proposed by Cheng *et al*. using real-time single cell analysis in macrophages expressing EGFP-tagged p65 and a TNFα promoter-driven mCherry. Following LPS stimulation, cells lacking TRIF show a pattern of NF-κB dynamics that is unaltered from wild-type cells, but activation of the TNFα promoter is impaired. In macrophages lacking MyD88, there is minimal NF-κB translocation to the nucleus in response to LPS stimulation, and there is no activation of the TNFα promoter. These findings confirm that signalling through MyD88 is the primary driver for LPS-dependent NF-κB translocation to the nucleus. The pattern of NF-κB dynamics in TRIF-deficient cells does not, however, directly reflect the kinetics of TNFα promoter activation, supporting the concept that TRIF-dependent signalling plays an important role in the transcription of this cytokine.

## Introduction

Pattern Recognition Receptor (PRR) detection of pathogens activates innate immune response in cells such as macrophages to control infections^1–3^. The PRR toll-like receptor 4 (TLR4) and its associated co-receptor myeloid differentiation factor 2 (MD-2) sense the Gram-negative bacterial outer membrane component lipopolysaccharide (LPS)^1, 4–7^. Following association with LPS, TLR4 is activated at the cell surface and recruits myeloid differentiation primary response gene 88 (MyD88) to its cytosolic Toll/IL-1R (TIR) domain via the adaptor protein MyD88 adaptor-like (Mal)^8–11^. TLR4 subsequently undergoes endocytosis in a CD14-dependent manner^12^, followed by recruitment of a second signalling adaptor protein TIR domain-containing adaptor-inducing interferon-beta (TRIF) via the TRIF-related adaptor molecule (TRAM)^13–16^. TLR4 signalling is transduced sequentially through the MyD88-dependent pathway from the cell membrane and the TRIF-dependent pathway from the endosome^14, 16^. There is a differential contribution of each pathway to inflammatory gene expression^17^, but how MyD88- and TRIF-dependent signalling impact on the dynamics of NF-κB translocation is unknown.

TLR4 signalling initiates translocation of the pro-inflammatory transcription factor nuclear factor kappa-light-chain enhancer of activated B cells (NF-κB) into the nucleus from the cytoplasm to induce gene transcription^18–20^. NF-κB in the nucleus is inactivated and exported out of the nucleus by the newly synthesized protein inhibitory κB (IκB)^21, 22^. The dynamics of NF-κB translocation in and out of the nucleus are thought to contribute to the pattern of inflammatory gene expression^22, 23^.

A recent study, coupling mathematical modeling to population-level functional analysis of MyD88-deficient and TRIF-deficient cells, suggested that LPS-driven activation of MyD88 in the absence of TRIF would alter NF-κB dynamics^24^. Using the prediction from their mathematical model, the authors propose that the variability of NF-κB dynamics in macrophages depends upon variability in TRIF signalling which determines the kinetics of tumor necrosis factor α (TNFα) production^24^. Single cell studies, however, have demonstrated that data from population-level experiments using bulk assays do not necessarily reflect events in individual cells because the heterogeneity of each individual cellular response is masked^22, 23, 25–29^.

Here, we studied the responses of wild-type, MyD88-deficient or TRIF-deficient single macrophages to LPS. Our work shows that, in contrast to the predictive model^24^, the pattern of NF-κB dynamics is unaltered in TRIF-deficient macrophages, but is delayed with an altered pattern in MyD88-deficient cells. Activation of a TNFα promoter was simultaneously monitored to assess the correlation between the pattern of NF-κB dynamics and TNFα expression. Unlike wild-type cells, TRIF-deficient macrophages show impaired activation of the TNFα promoter following LPS stimulation, despite having comparable NF-κB dynamics.

## Results

### Live cell imaging demonstrates that NF-κB oscillates in response to LPS in the absence of TRIF

**Figure 1:**
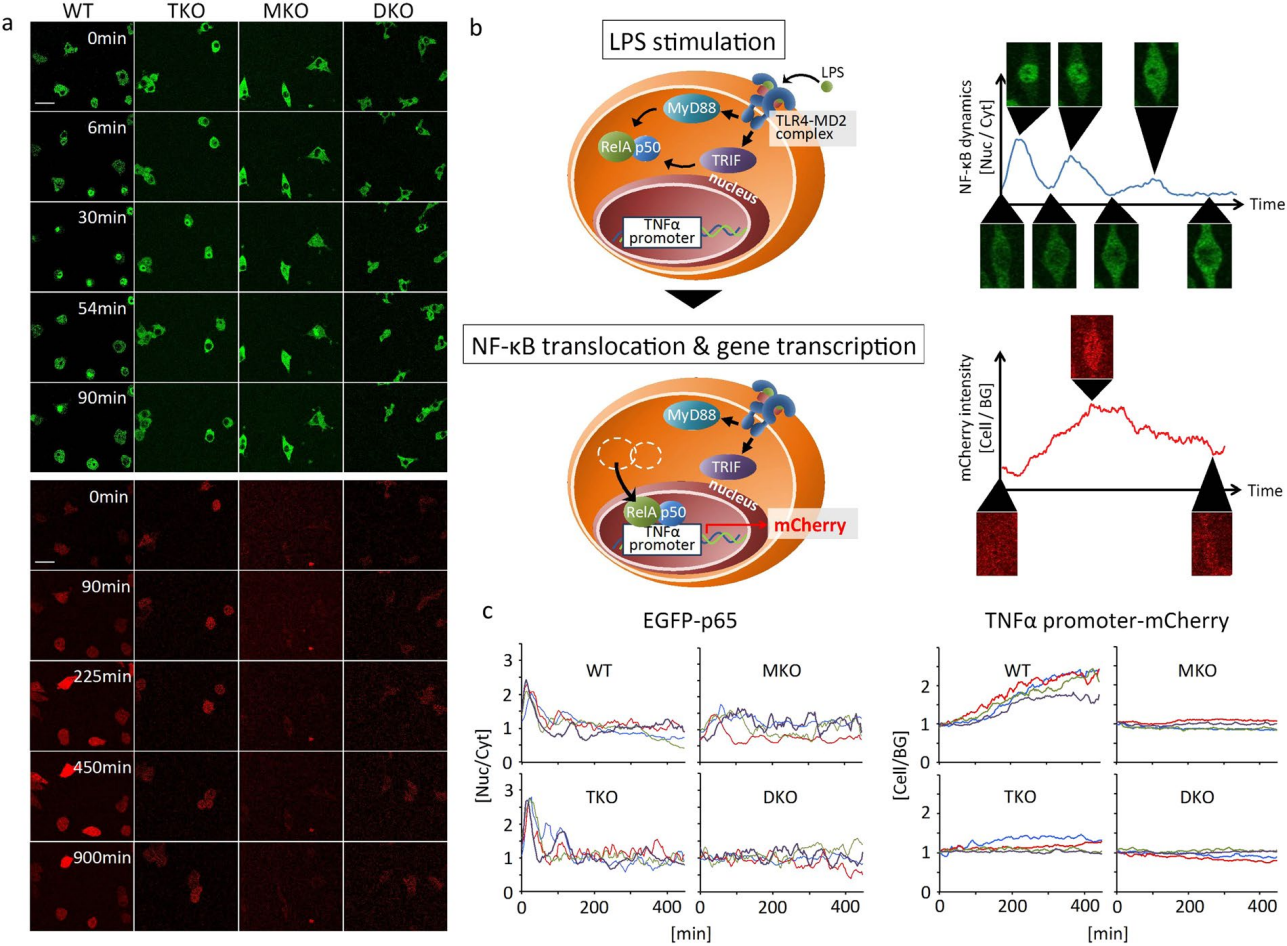
The different roles of MyD88- and TRIF-dependent pathways in LPS-induced NF-κB nuclear translocation and TNFα promoter activation. (**a**) Live cell imaging of EGFP-tagged NF-κB nuclear translocation (upper panels) and TNFα promoter-driven mCherry fluorescence (lower panels) in wild-type (WT), TRIF^−/−^ (TKO), MyD88^−/−^(MKO), and TRIF^−/−^/MyD88^−/−^ (DKO) in response to 500 ng/mL LPS. The scale bar indicates 50 μm. (**b**) A schematic diagram of TLR4 signaling to NF-κB and TNFα promoter activation in the reporter cells. TLR4 signal is mediated by MyD88- and TRIF-dependent pathways to activate NF-κB. The activated NF-κB translocates into the nucleus where it associates with the TNFα promoter, which induces mCherry gene transcription. (**c**) Time course of the ratio of NF-κB in the nucleus (Nuc) to those in the cytoplasm (Cyt) (left column), and the ratio of mCherry fluorescence intensity inside a cell (Cell) to those outside (background (BG)) (right column) in the reporter cells. Each figure shows four representatives of approximately 50 cells from four independent experiments. Individual cells are shown by coloured lines.

We set out to test the predictions of the mathematical model suggesting that NF-κB dynamics and TNFα production are impaired in LPS-dependent signalling in the absence of TRIF^24, 30^. We monitored NF-κB nuclear translocation and TNFα promoter activation in immortalized bone-marrow derived macrophage cell lines (iBMDM) from wild-type (WT), TRIF^−/−^ (TKO), MyD88^−/−^ (MKO), and TRIF^−/−^MyD88^−/−^ (DKO) mice by live cell confocal microscopy following LPS stimulation (Fig. 1a). These four lines of iBMDM stably express p65 fused with enhanced green fluorescent protein (EGFP) and generated mCherry driven from a TNFα promoter (Fig. 1b)^31^. The authenticity of the cell lines was verified by genotyping assays (Supplementary Fig. S1). NF-κB nuclear translocation following LPS stimulation was observed in all of the WT cells monitored in four independent experiments (Fig. 1a upper; Supplementary Movie 1). Single cell analysis (Supplementary Fig. S2) demonstrated that the oscillatory pattern of NF-κB dynamics consisted of a large initial peak followed by smaller secondary peaks in response to continuous LPS stimulation (Fig. 1c). TNFα promoter-driven mCherry fluorescence was simultaneously monitored (Fig. 1a lower; Supplementary Movie 2). The intensity of mCherry fluorescence gradually increased after LPS stimulation (Fig. 1c). The mCherry fluorescence reached a peak at approximately 450 minutes post-LPS stimulation and then decreased in most WT cells.

Next, we monitored NF-κB dynamics in TKO cells stimulated by LPS. Cheng *et al*. proposed that NF-κB is only activated by MyD88 so that in TKO cells there would be no second-phase NF-κB activity^24^. We found, however, that NF-κB oscillated in TKO in response to LPS (Fig. 1a upper; Supplementary Movie 4) with a pattern that appeared to be very similar to that seen in WT cells (Fig. 1c). The mCherry expression driven from the TNFα promoter did not increase in TKO following LPS stimulation, in contrast to WT cells (Fig. 1a lower and 1c right; Supplementary Movie 5). In LPS-stimulated MyD88^−/−^ cells, NF-κB translocation was delayed compared to WT cells and mCherry intensity did not increase (Fig. 1a,c; Supplementary Movie 7; Supplementary Movie 8). No NF-κB translocation or increase in mCherry intensity were observed in LPS-stimulated DKO (Fig. 1a upper; Supplementary Movie 10; Supplementary Movie 11). The slight fluctuations of NF-κB translocation in DKO were similar in the presence or absence of LPS stimulation (Fig. 1c), suggesting that LPS stimulation did not elicit NF-κB translocation in DKO cells.

### The timing of NF-κB nuclear translocation is the same in the presence or absence of TRIF in response to LPS

**Figure 2:**
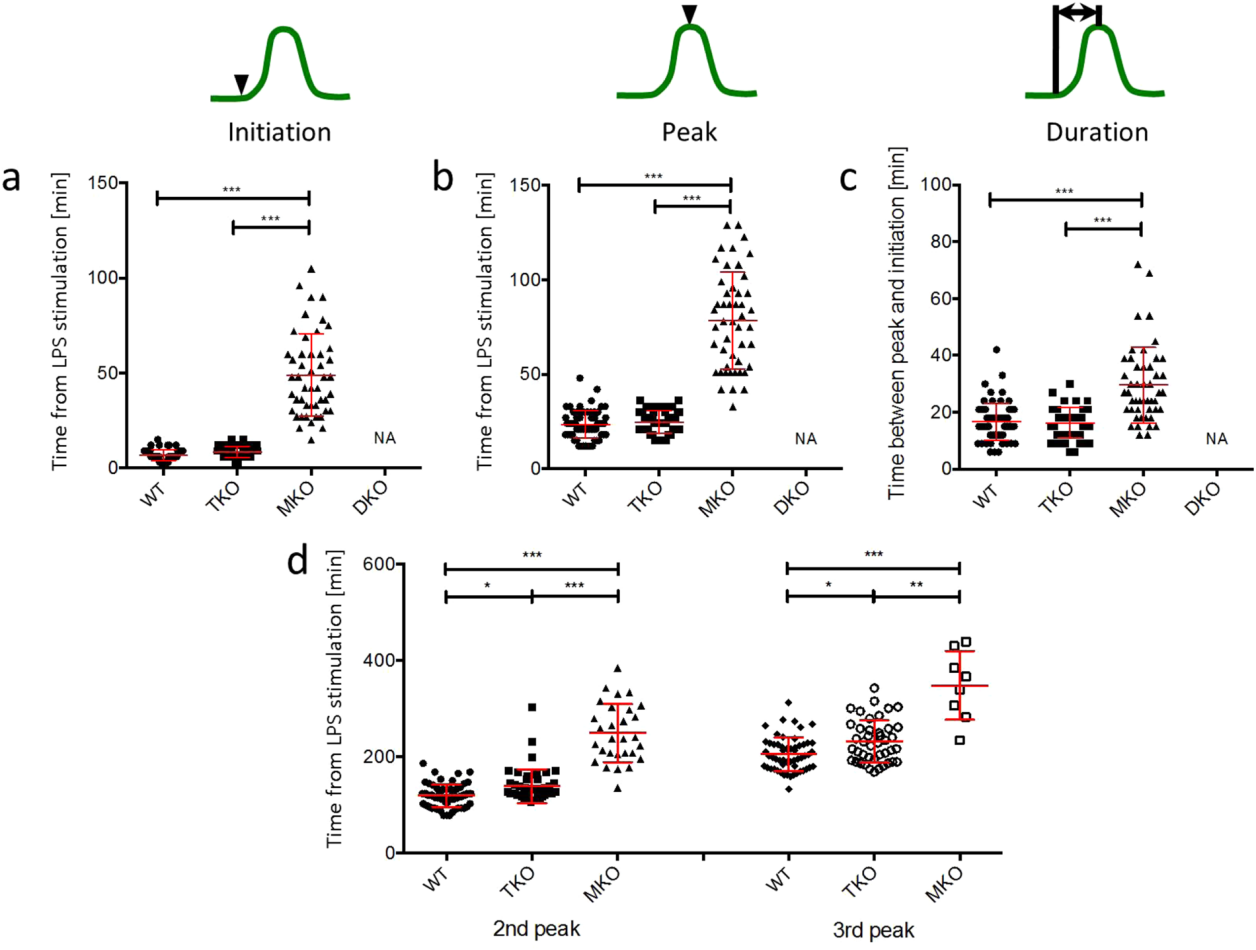
Timings of LPS-induced NF-κB nuclear translocation in TRIF-deficient macrophages are similar to those in wild-type macrophages. Population distributions of initiation timing (**a**), peak timing (**b**), and duration between initiation and peak timing (**c**) of the first peak in NF-κB nuclear translocation dynamics in the iBMDM reporter. Each dot shows a single cell from four independent experiments for each cell line. The bars show mean value ± s.d. (**d**) Peak timings of the second and third peaks in NF-κB nuclear translocation dynamics. ***P < 0.0001, **P < 0.01, *P < 0.05, one-way ANOVA with Kruskal-Wallis test.

We quantified the timing of NF-κB translocation in and out of the nucleus from single cell analysis of WT (n = 67), TKO (n = 50), and MKO (n = 48) (Fig. 2; Supplementary Table S1). Both WT and TKO initiated NF-κB nuclear translocation within 10 minutes after LPS stimulation, and there was no significant difference in initiation timing between the two cell types (p = 0.1476) (Fig. 2a; Supplementary Table S1). Initiation of the first peak in MKO was very heterogeneous with the mean initiation timing significantly delayed compared to WT and TKO (p < 0.0001) (Fig. 2a; Supplementary Table S1). The time taken to achieve the maximum fluorescent intensity for the first peak of NF-κB nuclear translocation were similar for WT and TKO cells (p > 0.9999) (Fig. 2b; Supplementary Table S1), but in the MKO it was significantly delayed (p < 0.0001) (Fig. 2b; Supplementary Table S1). To assess NF-κB nuclear occupancy during the first peak, we assumed a symmetrical peak because we did not observe a sustained nuclear occupancy after the highest point of the first peak. We thus calculated NF-κB nuclear duration time by subtracting the peak initiation time from the time the fluorescence achieving the maximum nuclear intensity (Fig. 2c; Supplementary Table S1). There was no significant difference in nuclear duration time between WT and TKO cells stimulated with LPS (p > 0.9999), while NF-κB occupied in the nucleus for a significantly longer period of time for MKO cells (p < 0.0001) (Fig. 2c; Supplementary Table S1).

Next, we quantified the timings of the secondary peaks of NF-κB translocation. We counted the peaks above noise level to be NF-κB nuclear translocations and confirmed that these peaks were genuine NF-κB nuclear translocations by inspecting the corresponding fluorescent live cell movies. A second peak was observed in 96% of WT and 94% of TKO with a third peak present in 84% of WT and 84% of TKO. The second and third peaks in TKO were slightly delayed compared to those in WT (Fig. 2d; Supplementary Table S1). The mean interval from the first peak to the second peak was longer in TKO than in WT (p = 0.0014), whereas there was no significant difference in the mean interval from the second peak to the third peak between WT and TKO (p = 0.27) (Supplementary Table S2). 70% of MKO cells showed a first peak of NF-κB translocation, and the second and third peaks were detected in 60% and 17% of cells with a first peak, respectively. The timings of both the second and third peaks in MKO were significantly later than those seen in LPS-stimulated WT and TKO (p < 0.0001) (Fig. 2d; Supplementary Table S1). The standard deviation of the second peak was greater than that of the first peak, and the standard deviation of the third peak was greater still in all the three strains (Supplementary Table S1), suggesting that the primary NF-κB peak is relatively synchronized between LPS-stimulated cells, but subsequent NF-κB translocation is more heterogeneous^32^.

### TRIF deficiency does not affect the magnitude of NF-κB nuclear translocation in macrophages in response to LPS

**Figure 3:**
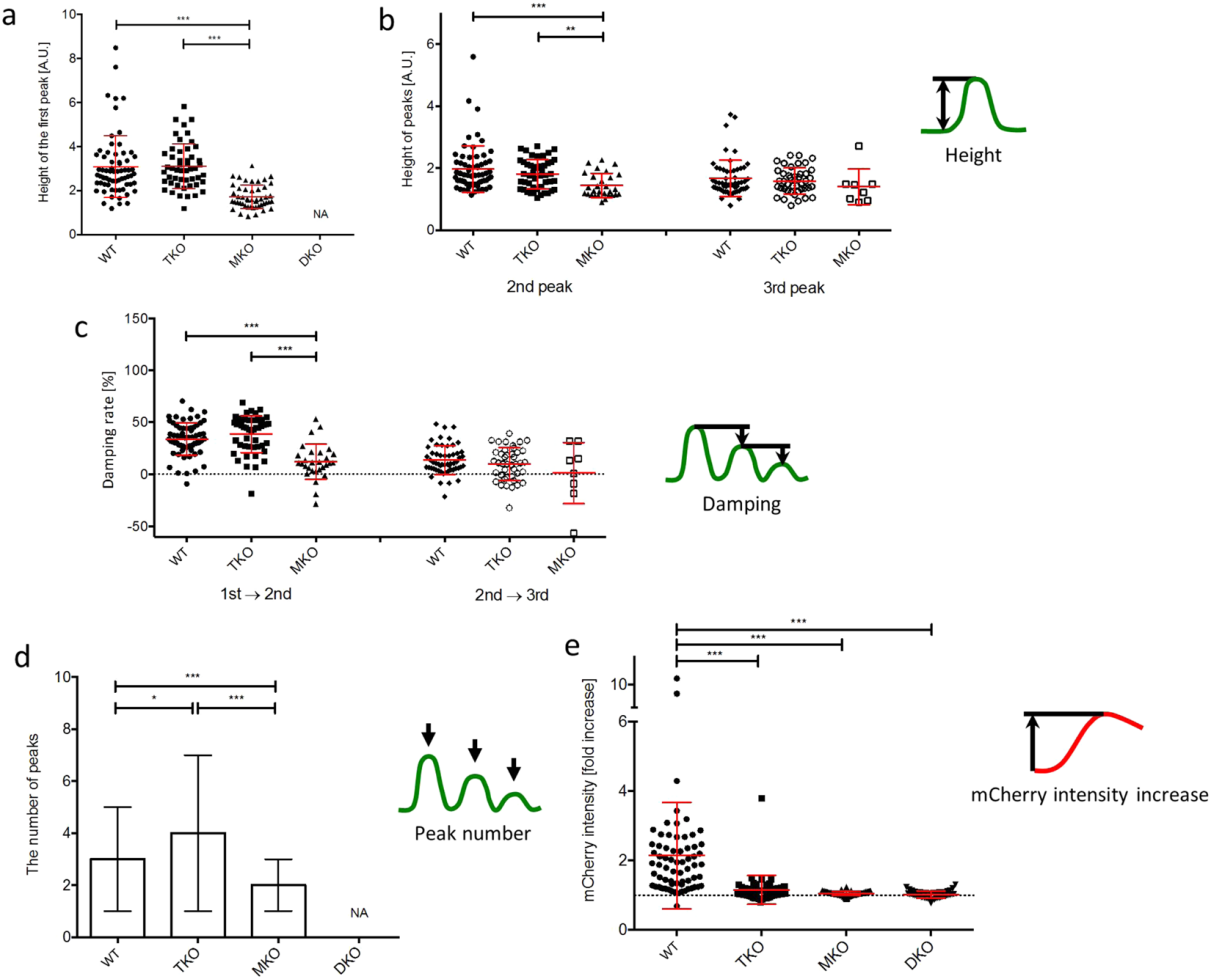
The magnitude of NF-κB nuclear translocation is not affected by the lack of TRIF-dependent signalling in response to LPS. The number of LPS-induced NF-κB oscillations does not decrease but TNFα promoter-driven mCherry expression is inhibited in TRIF-deficient macrophages in response to LPS. Each dot shows a single cell from four independent experiments for each cell line. The heights of the first peak (**a**), and the second and third peaks (**b**) of NF-κB translocation were determined by the value of the highest point in each peak of NF-κB translocation curve. (**c**) Damping rates indicate the decrease from the first peak to the second peak (1st → 2nd), and from the second peak to the third peak (2nd → 3rd). The bars show mean value ± s.d. ***P < 0.0001, **P < 0.01, one-way ANOVA with Kruskal-Wallis test. (**d**) The number of peaks in NF-κB nuclear translocation dynamics. Peaks above noise level that was determined by NF-κB curve in unstimulated cells were counted. The bars show median with range. (**e**) The fold increase of mCherry fluorescence intensity in response to LPS. Each dot shows a single cell from four independent experiments for each cell line. The bars show mean value ± s.d. The dot line indicates an one-fold (no increase). ***P < 0.0001, *P < 0.05, one-way ANOVA with Kruskal-Wallis test.

Robust NF-κB oscillation upon TNFα stimulation has been observed in several types of cells such as SK-N-AS cells^22^ and fibroblasts^27, 33^, but there is a progressively decreasing oscillation of NF-κB dynamics in the macrophage cell line RAW264.7^24, 31^ and in our iBMDM cells in response to LPS. We compared the heights of the first, second, and third peaks of NF-κB translocation to assess if TLR4 adaptor proteins are important for dampening the oscillatory patterns of NF-κB translocation. There were no significant differences in the heights of the first and second peaks between WT and TKO (p > 0.9999), but these peaks in MKO were significantly lower than WT and TKO (p < 0.0001) (Fig. 3a,b; Supplementary Table S3). Unlike the first and second peaks, there was no significant difference in the height of the third peak between these three cell types (p > 0.9999) (Fig. 3b right; Supplementary Table S3). Next, we compared the reduction rate (damping) of the height from the first peak to the second peak, and from the second peak to the third peak. Damping in TKO were comparable to those in WT from the first peak to the second peak (Fig. 3c left; Supplementary Table S4) and also from the second peak to the third peak (Fig. 3c right; Supplementary Table S4). Thus, any damping mechanism of NF-κB translocation oscillation is intact in the absence of TRIF. In MKO, however, whereas the first peak was significantly lower compared to WT and TKO, there was no significant damping. This implies that the damping mechanism is likely to specifically target MyD88-dependent signalling, or it may be activated in a MyD88-dependent manner.

### TRIF does not regulate the number of LPS-induced NF-κB oscillations, but is important for TNFα promoter activation

A correlation between the pattern of NF-κB dynamics and target gene expression has been suggested in a variety of cell types^22, 27, 32^, so we assessed the pattern of NF-κB oscillations in the various iBMDMs used in the study. The median number of peaks in WT cells was 3 peaks (range 1–5 peaks), in TKO was 4 peaks (range 1–7 peaks) whereas in MKO it was significantly less (median 2 peaks, range 1–3 peaks) (p < 0.0001) (Fig. 3d). This result shows that the pattern of LPS-induced NF-κB oscillations was not impaired by TRIF deficiency, but was clearly perturbed in the absence of MyD88. Next, we quantified TNFα promoter activation in response to LPS as the fold increase of mCherry fluorescence driven from the TNFα promoter. WT cells had a 2.1 ± 1.5 fold increase of mCherry fluorescence, whereas TKO showed only a 1.2 ± 0.4 fold increase (Fig. 3e; Supplementary Table S5). There was no increase in mCherry fluorescence in MKO or DKO in response to LPS stimulation. The impaired TNFα induction in TKO was also confirmed using flow cytometric analysis (Supplementary Fig. S3) since KDO2-Lipid A induces macrophage activation via the TLR4 signalling pathway in a similar manner to LPS^34^.

## Discussion

Here we show that the adaptor protein TRIF does not regulate the pattern of NF-κB translocation into and out of the nucleus in bone marrow derived macrophages. NF-κB plays a key role in LPS-induced inflammatory cytokine production^20^, and thus several studies have sought to correlate the pattern of NF-κB nuclear translocation dynamics with the kinetics of target gene expression^22, 24, 27, 31^. MyD88- and TRIF-dependent signalling are both important for driving LPS-induced TNFα production via NF-κB activation^16, 35–42^. No assessment has yet been made at the single cell level to show how MyD88- and TRIF-dependent signalling might correlate the NF-κB dynamics with target gene expression.

Cell population analysis which assesses a normalized cellular response in a population at a specific time point does not give any information about heterogeneity in NF-κB dynamics (Supplementary Fig. S4)^22, 23, 25–29^. Our data shows that the initial nuclear translocation of NF-κB after LPS stimulation is well synchronized between cells whereas the timing of secondary NF-κB translocations is very heterogeneous (Fig. 2; Supplementary Table S1). Other work investigating the dynamics of NF-κB translocation in a murine macrophage cell line used reporters stably expressed in RAW264.7 macrophages in response to LPS^24, 31^. We used RAW264.7 reporter cells to compare the signalling responses to, and validate the use of, the wild-type iBMDM reporter cells (Supplementary Fig. S5). No significant differences were seen in oscillatory patterns of NF-κB translocation in and out of the nucleus and TNFα promoter activation following LPS stimulation between the wild-type iBMDMs and RAW264.7 cells.

Mathematical modeling coupled to population cell analysis suggested that the patterns of LPS-induced NF-κB oscillation are regulated by TRIF signalling^24^. The model predicted that the absence of TRIF would not affect the primary peak of NF-κB translocation, but would suppress all secondary translocations of NF-κB in and out of the nucleus. Our work set out to test this model, but we found that the pattern of LPS-induced NF-κB oscillation in TRIF^−/−^ cells is similar to that observed in wild-type cells. Our results are consistent with previous reports that LPS-induced NF-κB translocation is mainly dependent on MyD88, but with little involvement of TRIF^8, 16, 18, 43^. In the absence of either TRIF or MyD88, we see little or no TNFα promoter activation respectively (Fig. 3e) suggesting that TNFα promoter activation is lost/reduced even when NF-κB oscillations are preserved. Hence our data suggests that some other feature of the system drives TNFα expression – either modification of NF-κB p65, that we cannot observe, or via some other distinct mechanism. For example, mitogen-activated protein kinases (MAPK) have an essential role in TLR4 ligand-induced TNFα production independent of NF-κB^44^. Rosadini *et al*. have shown that cytokine expression requires TRIF-dependent signalling to activate the MAPK-induced (activator protein 1) AP-1^45^. It is possible that there is interplay between NF-κB and AP-1 to generate efficient gene transcription^46, 47^. Our findings suggest that NF-κB nuclear translocation is essential but insufficient to activate *tnfα* gene expression. Activation of the TNFα promoter is likely, therefore, to require the cooperation of transcription factors distinctly activated by MyD88-dependent and TRIF-dependent signalling.

While NF-κB in TRIF-deficient macrophages was transported in and out of the nucleus in a similar pattern to that in wild-type cells, the 2nd and 3rd peaks in the NF-κB dynamics of TRIF-deficient cells were slightly but significantly delayed compared to those in wild-type cells (p = 0.0115 for the 2nd and 0.0161 for the 3rd peaks) (Fig. 2d). There have been few reports about the effects of delayed 2nd and 3rd peaks on gene expression. Timings of NF-κB nuclear translocation were shown to be out of phase with the cycle of IκBα replenishment^48^. We speculate that the delayed dynamics of NF-κB nuclear translocation can overlap with the peak of IκBα replenishment kinetics. This could result in faster interactions between NF-κB and IκBα in the nucleus, and thus NF-κB could be inactivated and shuttled out of the nucleus before it activates gene transcription.

We were not able to determine the source of the 2nd, 3rd and later phases of NF-κB activation in TRIF-deficient cells. Potentially there are different mechanisms that might explain the later peaks of NF-κB activation we see in LPS-stimulated TRIF-deficient cells. In TNFα- or LPS-stimulated primary mouse embryo fibroblasts (MEFs), autocrine/paracrine TNFα receptor signalling is important and it is possible that a similar ‘autocrine/paracrine’ mechanism explains MyD88/TRIF-independent signalling to activate the late phase of NF-κB translocation to and from the nucleus^29, 49^. LPS-stimulated wild-type MEFs show later initiation of NF-κB movement to and from the nucleus compared to iBMDMs, whereas TNFα-stimulated MEFs show rapid and oscillatory NF-κB nuclear translocation^29^. The difference in NF-κB response to LPS between iBMDMs and MEFs, however, may suggest that macrophages have different mechanisms that drive the late phase NF-κB activation in comparison to those used by MEFs, so autocrine/paracrine TNFα-dependent mechanism may not explain our data.

**Figure 4:**
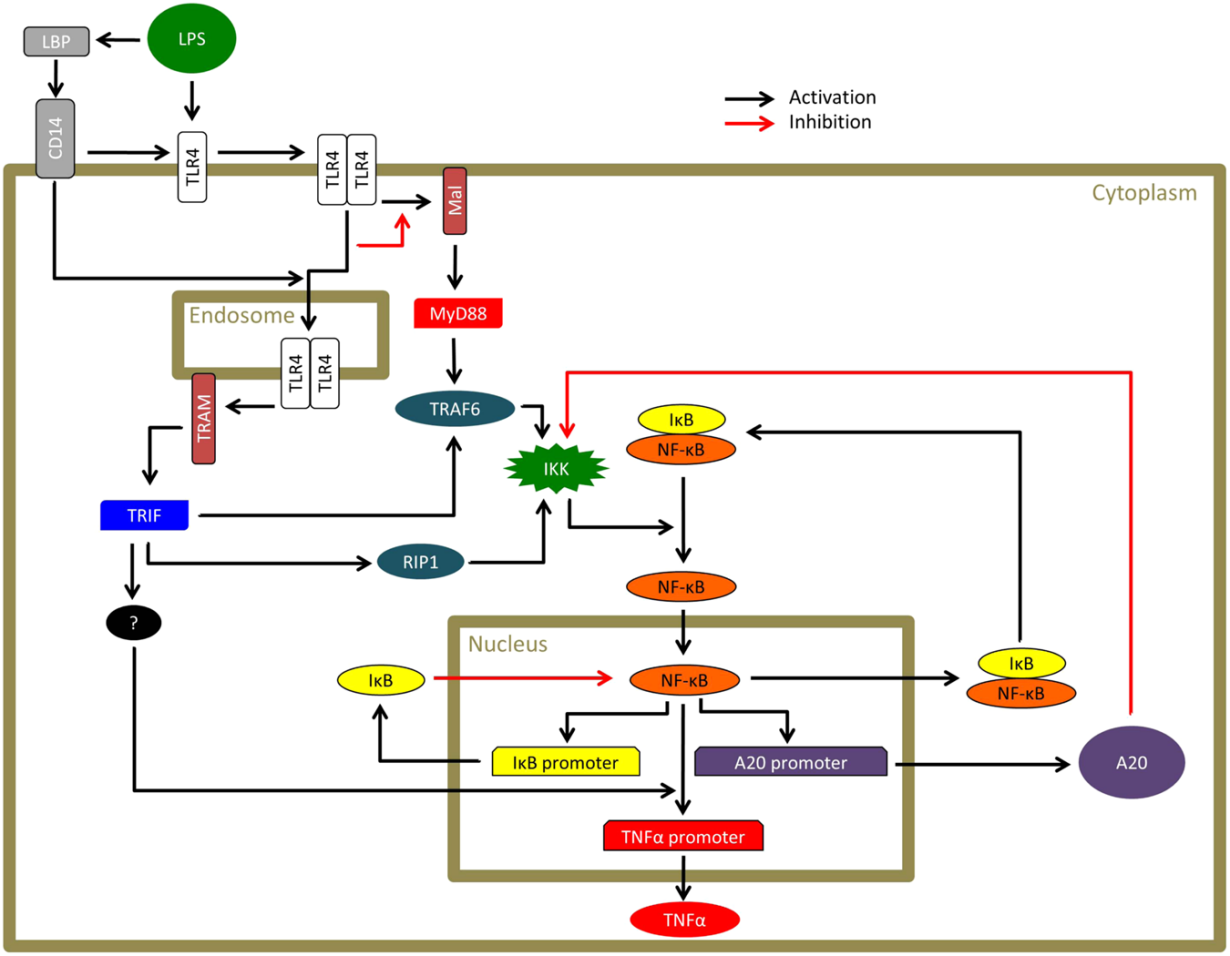
A representative schematic of the proposed TRIF contribution to NF-κB-dependent TNFα promoter activation. LPS activates TLR4 at the cell surface resulting in MyD88 activation via Mal. LPS also promotes TLR4 endocytosis following association with CD14 in the presence of the LPS-binding protein (LBP). TLR4 in the endosome subsequently recruits TRIF via TRAM. TLR4 signalling is transduced sequentially through the MyD88-dependent pathway from the cell membrane and the TRIF-dependent pathway from the endosome. Signals through MyD88-dependent and TRIF-dependent pathways distinctly activate IκB kinase (IKK) via the TNF receptor associated factor 6 (TRAF6) and the kinase receptor interacting protein 1 (RIP1). Activated IKK causes phosphorylation and degradation of IκB followed by NF-κB translocation to the nucleus. In the nucleus, NF-κB activates several promoters. NF-κB requires the cooperation of TRIF-dependent signalling to activate the TNFα promoter. NF-κB also activates promoters of negative regulators including IκB and A20. IκB inactivates NF-κB and leads it to shuttle out of the nucleus. A20 suppresses IKK through inhibition of the upstream signalling.

In summary, we propose that MyD88-dependent signalling plays a dominant role in LPS-induced NF-κB nuclear translocation, but with both MyD88 and TRIF-dependent signalling contributing to NF-κB-dependent *tnfα* gene transcription (Fig. 4). Comparison of our data with TNFα stimulation of fibroblasts shows some similarities in the patterns of NF-κB oscillation. In TNFα stimulated fibroblasts there is an interval of approximately 90 minutes between NF-κB translocation peaks^22, 27, 32, 33^ which is similar to what we observe in WT iBMDM (Supplementary Table S2). Considering the consistency of the periodic pattern in NF-κB dynamics between different types of cells in response to different stimuli, we speculate that the IκB-IKK feedback system may be mainly responsible for the pattern of NF-κB oscillatory dynamics regardless of the type of cell or the upstream signalling^33^. Macrophages, however, exhibit a large first peak followed by smaller secondary peaks in NF-κB oscillation (Fig. 1c)^24, 31^ in comparison to fibroblasts exhibiting sustained NF-κB oscillation^22, 27, 32, 33^. We speculate that macrophages may have an efficient negative feedback mechanism to suppress signalling upstream of the IκB-IKK system, in comparison to non immune cells, in order to prevent overproduction of cytokines and to protect the host from hyper-inflammatory responses. It is possible that some molecules upregulated by NF-κB could suppress IKK activation as a negative feedback loop. For example, A20 has been well studied for its negative regulatory role in IKK activation^23, 27, 28, 50^. A20, however, is unlikely to be the negative regulator here because it is not only expressed in macrophages but also in many other types of cells including fibroblasts^51^. Our single cell analysis underscores the importance of selecting the correct functional analyses to elucidate the correct general wiring of the network before developing an accurate mathematical models of signaling networks.

## Materials and Methods

### Cell culture

The iBMDM-derived reporter cell line cells and RAW264.7 cells which express enhanced green fluorescent protein (EGFP)-tagged p65 and TNFα promoter-driven mCherry were developed as described previously^31^. The cells were maintained in Dulbecco’s Modified Eagle’s Medium (DMEM; Sigma-Aldrich) supplemented with 10% (v/v) heat-inactivated fetal calf serum (FCS; Thermo Scientific), 2 mM L-glutamine (Sigma-Aldrich) and 20 mM HEPES (Sigma-Aldrich) at 37 °C, 5% CO_2_. Primary BMDM were isolated from tibiae and femurs of C57BL/6, MyD88^-/-37^or TRIF^-/-16^ mice and cultured in DMEM with 10%(v/v) FCS, 20% L929, 2 mM L-glutamine, and 100 U/mL penicillin and streptomycin at 37 °C, 5% CO_2_.

### Live cell microscopy

The cells were plated on a 35-mm glass-bottom dish (Greiner Bio-One, #627860) at a concentration of 1.0 × 10^5^ cells in phenol red-free DMEM (Sigma-Aldrich) supplemented with 10% (v/v) FCS, 2 mM L-glutamine and 20 mM HEPES, and allowed to settle for 8 hours prior to experiments. The dish was mounted on the stage of a confocal microscope (TCS SP5, Leica) and kept at 37 °C, 5% CO_2_ during the experiment in a climate chamber. Live cell imaging was performed immediately after stimulating the cells with LPS (Enzo Life Sciences). Images were sequentially captured through a 40× oil-immersion objective (NA1.25) with 2.0x zoom every 3 minutes for 15 hours. EGFP was excited by argon laser at 488 nm with 15% laser power and mCherry by He-Ne laser at 594 nm with 8% laser power. The emission signals from EGFP and mCherry were collected with 504–565 nm band pass and 604–656 nm band pass, respectively. We also recorded the transmitted beam as a bright field image. The image dimension used was 512 × 512 pixels. Scan speed was 100 lines per second, and 3 line averaging was applied. The pinhole size was 1 A.U., and the thickness of the focal plane was 0.96 μm. Acquired images were exported as 16-bit TIFF files for MATLAB-based automated analysis.

### MATLAB-based automated single cell analysis

A custom-built MATLAB script was used to automatically assess NF-κB nuclear translocation and TNFα promoter-driven mCherry expression (Supplementary Fig. S2﻿; the code can be accessed at http://dx.doi.org/10.17863/CAM.6018). Segmentation to determine the area of a cell body was performed based on information from both the EGFP and bright field images. A nuclear region was determined as a circle with a diameter which was selected manually. The area of a cell body was determined from the bright field images. A cytosolic area was defined as the area remaining after subtracting the nuclear area from the area of a cell body. The EGFP intensity of all pixels in the nucleus and the cytoplasm was measured and then averaged for the nuclear value and the cytosolic value in each frame. The ratio of the nuclear value to the cytosolic value was calculated in each frame and then averaged by ten frames to make a NF-κB nuclear translocation curve. The NF-κB noise level was determined from fluctuations in NF-κB dynamics seen in unstimulated cells. Peaks above noise level were considered to be ones induced by NF-κB nuclear translocation. The intensity of mCherry fluorescence was measured from the area of a cell body determined by bright field images and then averaged for the cell body. Background noise was measured from an area without a cell and then averaged. This automated analysis only works for a single cell which is not next to another cell for the entire duration of movie. This is because the image analysis system is unable to distinguish two adjacent cells as being different from a single cell. We therefore excluded any cell that touches another cell or that divides during the course of imaging from the data analysis.

### Flow cytometric analysis for TNFα induction

Primary macrophages for this analysis were prepared from murine bone marrow. Briefly, mice were maintained in specific pathogen free condition and all procedures were approved by the NIAID Animal Care and Use Committee (National Institute of Health, Bethesda, MD). Bone marrow progenitors were differentiated into BMDM during a 6-day culture in complete DMEM supplemented with 30 ng/mL recombinant mouse M-CSF (R&D systems), added at days 0 and 3. BMDM were re-plated on day 6 into 48-well plates and stimulated on day 7 with Kdo2-Lipid A (KLA; Avanti Polar Lipids), with addition of Brefeldin A (BD Goldi Plug). Anti-mouse TNFα antibody, clone MP6-XT22 (BioLegend) was used. The data were collected with a BD LSRFortessa and analyzed in FlowJo software.

### Genotyping

DNA was extracted from the iBMDM reporter cell lines using a DNeasy Blood & Tissue Kit (Qiagen), and amplified by PCR using primers: MyD88 forward (5′-AGCCTCTACACCCTTCTCTTCTCCACA-3′), MyD88 reverse (5′-AGACAGGCTGAGTGCAAACTTGTGCTG-3′), MyD88 neomycin (5′-ATCGCCTTCTATCGCCTTCTTGACGAG-3′), TRIF forward (5′-CTGACACACTGTGTACTTACTAGGTGC-3′), TRIF reverse (5′-CAAGATTGGACTTCACCTGGGTCCTTA-3′), and TRIF neomycin (5′-CTAAAGCGCATGCTCCAGACTGCCTTG-3′). The resulting PCR products were analyzed by electrophoresis in 1% agarose gel with SYBR® Safe DNA gel stain (Invitrogen) at 100 volts.

### Western blot analysis

Following stimulation with LPS (Enzo Life Sciences), Cells were washed with cold PBS, gently scraped, and resuspended in lysis buffer (1.0% NP-40, 150 mM sodium chloride, 50 mM Tris, pH8.0) containing proteinase inhibitor cocktail (Sigma-Aldrich) and phosphatase inhibitor cocktail 2 and 3 (Sigma-Aldrich). Protein samples were mixed with laemmli sample buffer (Bio-Rad), resolved by 12% SDS-PAGE gel, and transferred to nitrocellulose membranes (Millipore). The membrane was probed by phospho-NF-κB p65 (Ser536) rabbit monoclonal antibody (Cell signaling, #3033; 1:1000 dilution), NF-κB p65 rabbit polyclonal antibody (Santa Cruz, #sc-372; 1:1000 dilution), Phospho-IRF3 (Ser396) rabbit monoclonal antibody (Cell signaling, #4947; 1:1000 dilution), IRF3 rabbit monoclonal antibody (Cell signaling, #4302; 1:1000 dilution), IκBα rabbit polyclonal antibody (Santa Cruz, #sc-371; 1:1000 dilution), and α-actin goat polyclonal antibody (Santa Cruz, #sc-1616; 1:1000 dilution).

### Statistical analysis

All statistical analyses were performed using Prism 6.0 software (GraphPad Prism). One-way analysis of variance (ANOVA) with Kruskal-Wallis test was followed by Dunn’s multiple comparison test. *P* < *0.05* was considered as significant difference.

## Electronic supplementary material

Supplementary Information

Supplementary movie 1

Supplementary movie 2

Supplementary movie 3

Supplementary movie 4

Supplementary movie 5

Supplementary movie 6

Supplementary movie 7

Supplementary movie 8

Supplementary movie 9

Supplementary movie 10

Supplementary movie 11

Supplementary movie 12
