# Supplementary material for: Lipopolysaccharide-induced NF-κB nuclear translocation is primarily dependent on MyD88, but TNFα expression requires TRIF and MyD88
Source: Sci Rep. 2017 May 3;7:1428. doi: 10.1038/s41598-017-01600-y (PMC5431130; doi:10.1038/s41598-017-01600-y)
Supplement: Supplementary file 1 — Supplementary Information [file 41598_2017_1600_MOESM1_ESM.pdf]

Supplementary Information

Lipopolysaccharide-induced NF-κB nuclear translocation is primarily dependent on MyD88, but TNFα expression requires TRIF and MyD88

Jiro Sakai[1], Eugenia Cammarota[2], John A. Wright[1], Pietro Cicuta[2], Rachel Gottschalk[3], Ning Li[3], Iain D. C. Fraser[3] and *Clare E. Bryant[1]

[1]Department of Veterinary Medicine, University of Cambridge, Cambridge CB3 0ES, United Kingdom

[2]Sector of Biological and Soft Systems, Cavendish Laboratory, University of Cambridge, Cambridge CB3 0HE, United Kingdom

[3]Laboratory of Systems Biology, National Institute of Allergy and Infectious Diseases, National Institute of Heath, Bethesda, MD 20892, USA

Correspondence and requests for materials should be addressed to C.E.B. (email: ceb27@cam.ac.uk).

Supplementary Movie 1

This movie shows EGFP-tagged RelA (p65) in WT stimulated by 500ng/mL LPS. The time-lapse covers a period of 15 hours, with 30 minutes elapsed time per second of movie (QuickTime; 1168.49 KB).

Supplementary Movie 2

This movie shows mCherry driven from TNFα promoter in WT stimulated by 500ng/mL LPS. The time-lapse covers a period of 15 hours, with 30 minutes elapsed time per second of movie (QuickTime; 497.14 KB).

Supplementary Movie 3

This movie shows bright field of WT stimulated by 500ng/mL LPS. The time-lapse covers a period of 15 hours, with 30 minutes elapsed time per second of movie (QuickTime; 1942.2 KB).

Supplementary Movie 4

This movie shows EGFP-tagged RelA (p65) in TKO stimulated by 500ng/mL LPS. The time-lapse covers a period of 15 hours, with 30 minutes elapsed time per second of movie (QuickTime; 1077.12 KB).

Supplementary Movie 5

This movie shows mCherry driven from TNFα promoter in TKO stimulated by 500ng/mL LPS. The time-lapse covers a period of 15 hours, with 30 minutes elapsed time per second of movie (QuickTime; 166.22 KB).

Supplementary Movie 6

This movie shows bright field of TKO stimulated by 500ng/mL LPS. The time-lapse covers a period of 15 hours, with 30 minutes elapsed time per second of movie (QuickTime; 1783.54 KB).

Supplementary Movie 7

This movie shows EGFP-tagged RelA (p65) in MKO stimulated by 500ng/mL LPS. The time-lapse covers a period of 15 hours, with 30 minutes elapsed time per second of movie (QuickTime; 1221.69 KB).

Supplementary Movie 8

This movie shows mCherry driven from TNFα promoter in MKO stimulated by 500ng/mL LPS. The time-lapse covers a period of 15 hours, with 30 minutes elapsed time per second of movie (QuickTime; 96.67 KB).

Supplementary Movie 9

This movie shows bright field of MKO stimulated by 500ng/mL LPS. The time-lapse covers a period of 15 hours, with 30 minutes elapsed time per second of movie (QuickTime; 1764.43 KB).

Supplementary Movie 10

This movie shows EGFP-tagged RelA (p65) in DKO stimulated by 500ng/mL LPS. The time-lapse covers a period of 15 hours, with 30 minutes elapsed time per second of movie (QuickTime; 1884.65 KB).

Supplementary Movie 11

This movie shows mCherry driven from TNFα promoter in DKO stimulated by 500ng/mL LPS. The time-lapse covers a period of 15 hours, with 30 minutes elapsed time per second of movie (QuickTime; 85.92 KB).

Supplementary Movie 12

This movie shows bright field of DKO stimulated by 500ng/mL LPS. The time-lapse covers a period of 15 hours, with 30 minutes elapsed time per second of movie (QuickTime; 1958.66 KB).

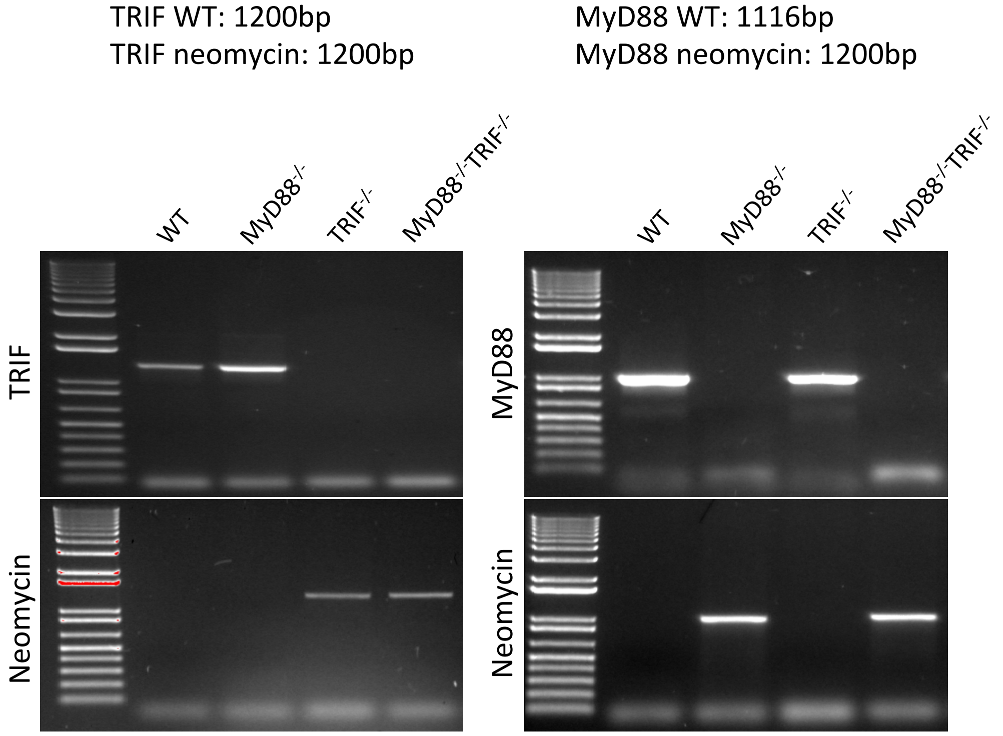

Supplementary Figure S1 Genotyping assay to verify the authenticity of the cell lines

Following DNA amplification with PCR, samples were detected by electrophoresis in a 1% agarose gel. The TRIF and MKO genes were assessed as a positive control, and the neomycin resistant gene was assessed as a negative control.

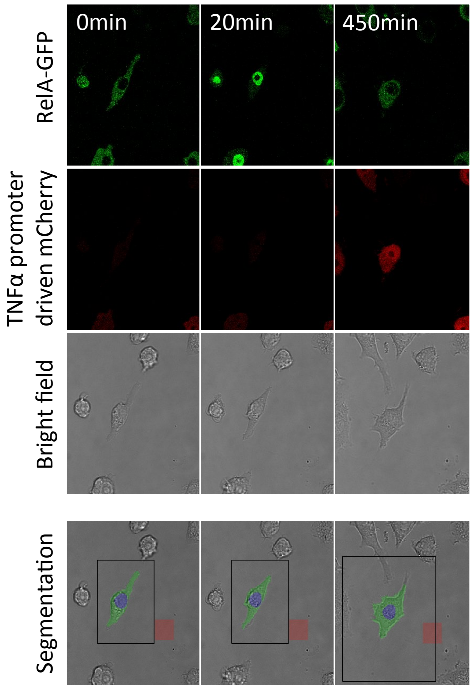

Blue: Nucleus (Nuc), Green: Cytoplasm (Cyt),
Red: Background (BG)

Supplementary Figure S2 MATLAB-based automated analysis of NF-κB translocation and mCherry fluorescence intensity

Segmentation to determine the area of a cell body was performed based on EGFP pictures and bright field pictures. A nuclear region was determined as a circle with a diameter which was selected manually (blue region). In this study, the size of a nuclear area was assumed to be constant. The area of a cell body was determined from the bright field pictures. A cytosolic area was defined as the area remaining after subtracting the nuclear area from the area of a cell body (green areas). The EGFP intensity of all pixels in the nucleus or the cytoplasm was measured and then averaged for the nuclear value (Nuc) and the cytosolic value (Cyt) in each frame. NF-κB nuclear translocation was assessed by the ratio of the nuclear EGFP intensity to the cytoplasm (Nuc/Cyt). The intensity of

mCherry fluorescence was measured from the area of a cell body determined by bright field pictures and then averaged for the cell body (Cell). Background noise (BG) was measured from a rectangular area without a cell (red areas) and then averaged. The intensity of mCherry fluorescence in the cell body was normalized by background noise (Cell/BG).

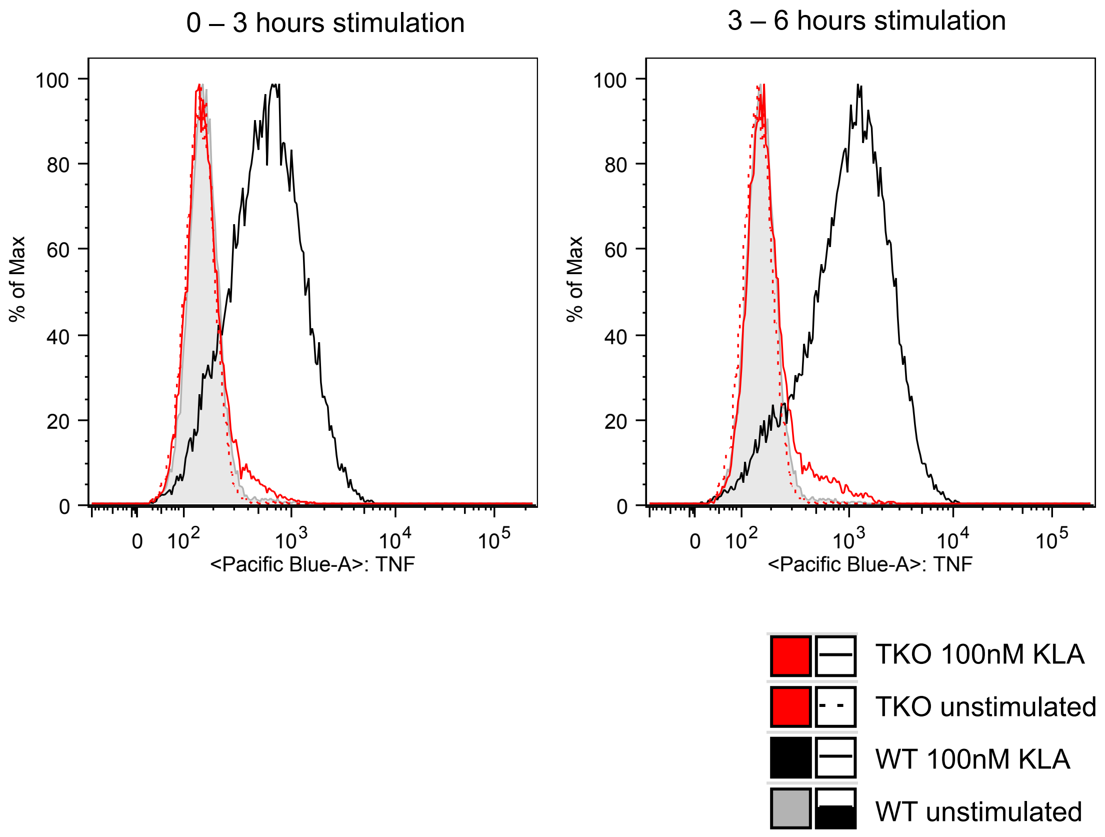

Supplemental figure S3 LPS-induced TNFα induction was impaired in macrophages from TRIF$^{-/-}$ mice.

Primary BMDM derived from wild-type C57BL/6 or TRIF$^{-/-}$ mice were stimulated with 100nM Kdo2-Lipid A (KLA) for either 3 hours or 6 hours, as indicated. Intracellular TNFα in BMDM were stained and analyzed by flow cytometry. Data are representative of two independent experiments.

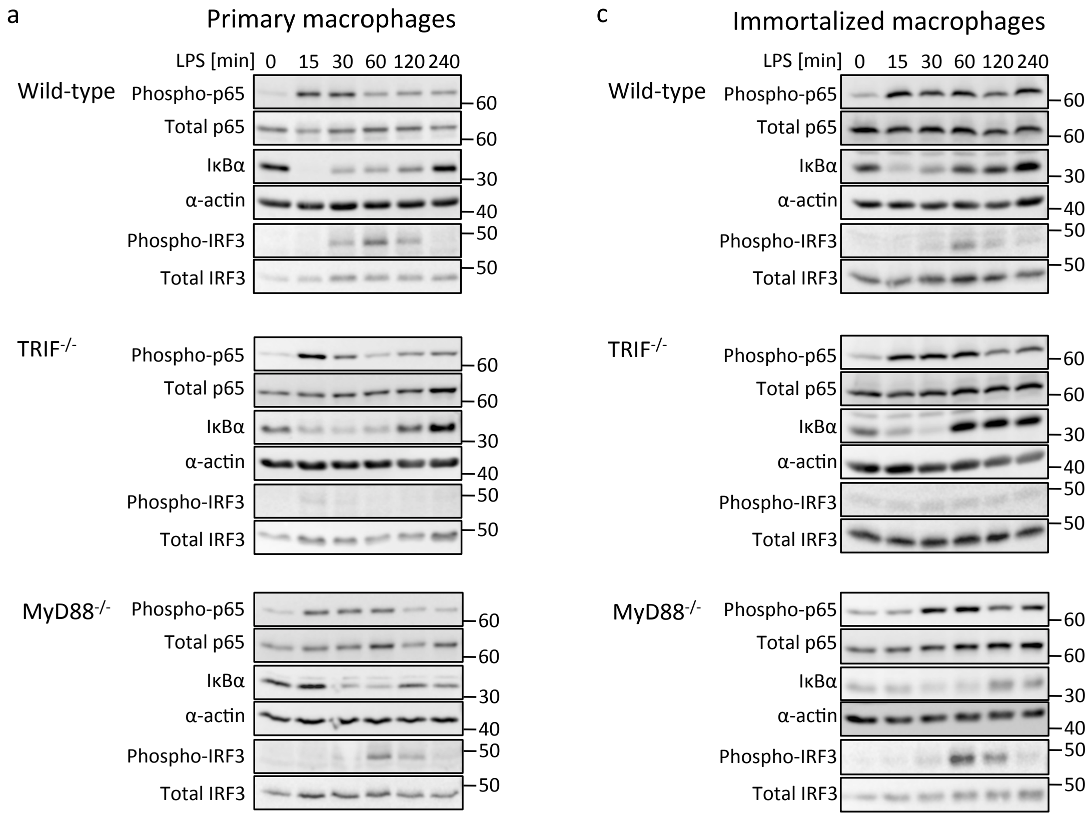

Supplemental figure S4 Cell population assay shows an averaged response of a population, but does not necessarily reflect individual cellular responses.

(a) Western blotting of p65 phosphorylation, IκBα degradation, and IRF3 phosphorylation in primary BMDMs (a) and immortalized BMDMs (b) from wild-type, TRIF$^{-/-}$, and MyD88$^{-/-}$ mice following 1μg/mL LPS stimulation.

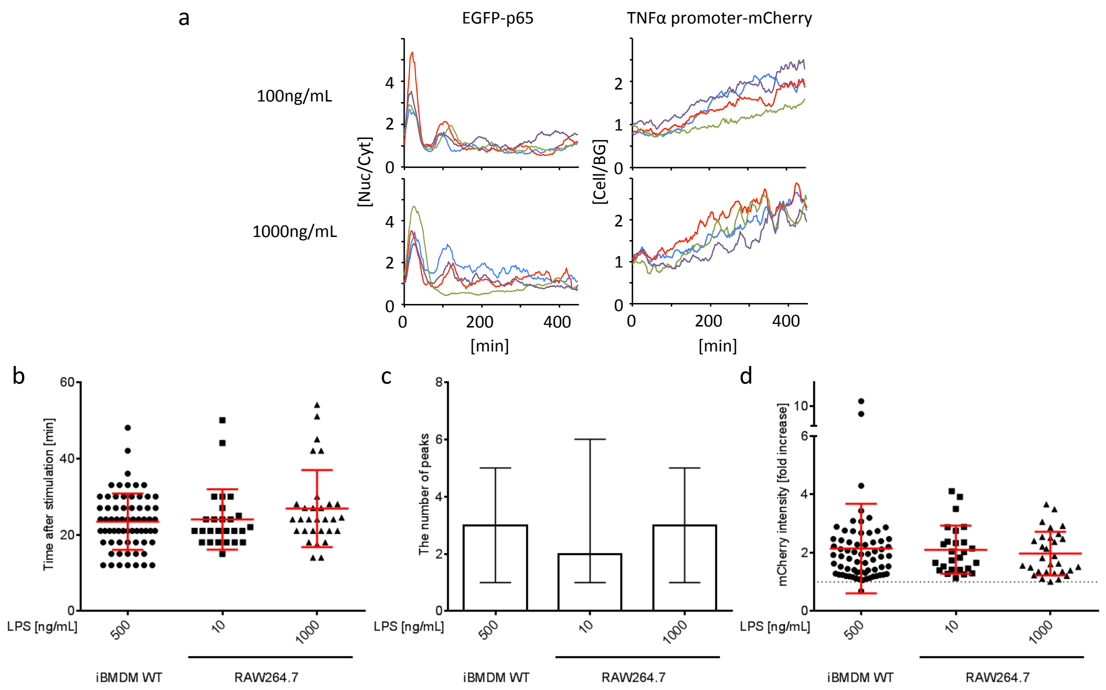

Supplemental figure S5 Wild-type iBMDMs demonstrate similar NF-κB nuclear translocation dynamics and TNFα promoter activation to RAW264.7 cells in response to LPS.

(a) Time course of the ratio of NF-κB (left column), and the ratio of mCherry fluorescence intensity (right column) in the RAW264.7 cells in response to 100 or 1000ng/mL LPS. Each figure shows four representatives of approximately 30 cells from four independent experiments. Individual cells are shown by coloured lines. (b) Comparison of peak timing of the first peak in NF-κB nuclear translocation dynamics between the iBMDM and the RAW264.7 cells. The bars show mean value ± s.d. One-way ANOVA with Kruskal-Wallis test shows no significant difference between the iBMDM and the RAW264.7 cells. (C) Comparison of the number of peaks in NF-κB nuclear translocation dynamics between the iBMDM and the RAW264.7 cells. The bars show median with range. One-way ANOVA with Kruskal-Wallis test shows no significant difference

between the iBMDM and the RAW264.7 cells. (d) Comparison of the fold increase of mCherry fluorescence intensity between the iBMDM and the RAW264.7 cells. The bars show mean value ± s.d. The dot line indicates an one-fold (no increase). One-way ANOVA with Kruskal-Wallis test shows no significant difference between the iBMDM and the RAW264.7 cells.

Supplementary Table S1 Timings of NF-κB peaks

500ng/mL LPS
(minutes; mean±s.d.)

	First peak			Second peak	Third peak
	Initiation time	Peak time	Duration	Peak time	Peak time
WT (n=67)	6.8±2.8	23.4±7.4	16.6±6.5	118.6±23.1	204.8±35.1
TKO (n=50)	8.5±3.0	24.7±5.9	16.3±5.4	138.4±34.8	231.4±44.2
MKO (n=48)	48.9±21.7	78.5±25.5	29.6±13.3	248.7±60.3	347.3±71.2

Supplementary Table S2 Mean interval between consecutive peaks

500ng/mL LPS
(minutes; mean±s.d.)

	2nd – 1st	3rd – 2nd
WT (n=67)	95.3±23.6	89.1±30.7
TKO (n=50)	113.9±32.7	97.1±38.6
MKO (n=48)	173.5±57.7	137.6±78.9

Supplementary Table S3 Heights of NF-κB peaks

500ng/mL LPS
(A.U.; mean±s.d.)

	First peak	Second peak	Third peak
WT (n=67)	3.1±1.4	1.9±0.8	1.7±0.6
TKO (n=50)	3.1±1.0	1.8±0.5	1.6±0.4
MKO (n=48)	1.7±0.5	1.4±0.4	1.4±0.6

Supplementary Table S4 Reduction rates of the height between consecutive peaks (Damping)

500ng/mL LPS
(%.; mean±s.d.)

	Damping 1st ➔2nd	Damping 2nd ➔3rd
WT (n=67)	33.8±15.9	13.6±13.8
TKO (n=50)	38.6±17.9	9.9±15.7
MKO (n=48)	12.0±16.8	1.0±29.4

Supplementary Table S5 Fold-increase of mCherry fluorescence intensity

500ng/mL LPS
(fold increase; mean±s.d.)

WT (n=67)	2.1±1.5
TKO (n=50)	1.2±0.4
MKO (n=48)	1.1±0.1
DKO (n=48)	1.0±0.1
